# Development of SDS-Modified PbO_2_ Anode Material Based on Ti^3+^ Self-Doping Black TiO_2_NTs Substrate as a Conductive Interlayer for Enhanced Electrocatalytic Oxidation of Methylene Blue

**DOI:** 10.3390/molecules28196993

**Published:** 2023-10-09

**Authors:** Mai Xu, Chunli Gao, Xiaoyan Zhang, Xian Liang, Yunhu Hu, Fengwu Wang

**Affiliations:** 1 School of Chemistry and Material Engineering, Huainan Normal University, Huainan 232038, China; xumai@hnnu.edu.cn (M.X.); xumai2215@126.com (X.Z.); liangxian226@163.com (X.L.); 2 School of Materials Science and Engineering, Anhui University of Science and Technology, Huainan 232001, China; xumai2215@163.com

**Keywords:** Ti^3+^ self-doping black TiO_2_ nanotube arrays, PbO_2_ electrode, twelve sodium dodecyl sulfate (SDS), electrocatalytic oxidation (EO), wastewater treatment

## Abstract

Efficient and stable electrode materials are urgently required for wastewater treatment in the electrocatalytic degradation of toxic and refractory organic pollutants. Ti^3+^ self-doping black TiO_2_ nanotube arrays (Ti/B-TiO_2_-NTs) as an interlayer were used for preparing a novel PbO_2_ electrode via an electrochemical reduction technology, and a sodium dodecyl sulfate (SDS)-modified PbO_2_ catalytic layer was successfully achieved via an electrochemical deposition technology. The physicochemical characterization tests showed that the Ti/B-TiO_2_-NTs/PbO_2_-SDS electrodes have a denser surface and finer grain size with the introduction of Ti^3+^ in the interlayer of Ti/TiO_2_-NTs and the addition of SDS in the active layer of PbO_2_. The electrochemical characterization results showed that the Ti^3+^ self-doping black Ti/TiO_2_-NTs/PbO_2_-SDS electrode had higher oxygen evolution potential (2.11 V vs. SCE), higher electrode stability, smaller charge-transfer resistance (6.74 Ω cm^−2^), and higher hydroxyl radical production activity, leading to it possessing better electrocatalytic properties. The above results indicated that the physicochemical and electrochemical characterization of the PbO_2_ electrode were all enhanced significantly with the introduction of Ti^3+^ and SDS. Furthermore, the Ti/B-TiO_2_-NTs/PbO_2_-SDS electrodes displayed the best performance on the degradation of methylene blue (MB) in simulated wastewater via bulk electrolysis. The removal efficiency of MB and the chemical oxygen demand (COD) could reach about 99.7% and 80.6% under the optimal conditions after 120 min, respectively. The pseudo-first-order kinetic constant of the Ti/B-TiO_2_-NTs/PbO_2_-SDS electrode was 0.03956 min^−1^, which was approximately 3.18 times faster than that of the Ti/TiO_2_-NTs/PbO_2_ electrode (0.01254 min^−1^). In addition, the Ti/B-TiO_2_-NTs/PbO_2_-SDS electrodes showed excellent stability and reusability. The degradation mechanism of MB was explored via the experimental identification of intermediates. In summary, the Ti^3+^ self-doping black Ti/TiO_2_-NTs/PbO_2_-SDS electrode is a promising electrode in treating wastewater.

## 1. Introduction

The harmonious coexistence between humans and nature has always been the ultimate aspiration of mankind. However, the excessive production of non-degradable organic pollutants produced by human beings is putting enormous pressure on the environment [1,2]. Textile wastewater containing persistent and toxic carcinogens such as methyl orange, rhodamine B, Congo red, and methylene blue (MB) is persistent and toxic carcinogens pose significant threats to both the aquatic environment and human health [3]. Therefore, implementing effective strategies for the removal of organic dyes from wastewater is related to the relationship between the sustainable development of society and nature.

Recently, several technologies have been developed for the removal of textile-dyeing wastewater. However, the desired effect cannot be achieved due to the poor mineralization effect in the treatment via conventional treatment methods [4]. Therefore, it is imperative to develop a clean, energy-saving, versatile, and efficient new approach for wastewater treatment.

The electrochemical oxidation (EO) process, as a promising method for degrading organic industrial effluents, has attracted growing interest nowadays due to its high efficiency, simple operation, and environmentally friendly technology [4,5]. For the electrochemical method, the anodic material is a crucial factor because it determines the effectiveness of mechanisms and reaction pathways of electro-catalytic oxidation [6]. Accordingly, developing a high-catalytic-efficiency and stable electrode is essential for enhancing the sewage treatment capacity.

Over the last few decades, various electrode materials have been reported for mineralizing refractory pollutants, including RuO_2_, IrO_2_, boron-doped diamond (BDD), and SnO_2_ electrodes [7,8]. Among them, the PbO_2_ electrode, known for its moderate price, simple preparation, high electrocatalytic activity, and good corrosion resistance, has been widely used as an anode material in the field of electrochemical water treatment [9]. However, traditional PbO_2_-coated anodes still have some disadvantages, such as a small specific surface area, large internal stress, and easy deactivation and brittleness [10]. A PbO_2_ electrode is usually easy to peel off from a flat Ti substrate because of the large internal stress, resulting in the leakage of the Pb element into the environment [11].

To improve the service life and electrocatalytic properties of the PbO_2_-based anodes, various strategies have been exploited by doping metal or non-metallic ions, performing substrate modification, using a nanostructured composite, and introducing an intermediate layer [12].

Anodically formed vertically oriented TiO_2_ nanotube arrays (Ti/TiO_2_-NTs) have also been extensively utilized as the catalyst or the middle layer in (photo-) electrochemical advanced oxidation processes for wastewater treatment [13].

Nevertheless, the semiconductive properties of Ti/TiO_2_-NTs impeded their further use in electrochemistry. To solve this problem, electrochemical self-doping via electrochemical reduction under mild conditions has been employed to improve the semiconducting behavior of Ti/TiO_2_-NTs. In this method, electrochemical reduction generates Ti^3+^, which increases the electrical conductivity of Ti/TiO_2_-NTs and creates more active sites [14].

Therefore, doping the interlayer with Ti^3+^ is supposed to significantly improve the electron-transfer efficiency between the PbO_2_ coating layer and the Ti substrate, hence improving the electrocatalytic activity and stability of the PbO_2_ anode for the electrochemical degradation of refractory organic pollutants.

To enhance both the electrocatalytic performance and stability of the PbO_2_ electrode, a novel Ti^3+^ self-doping black Ti/TiO_2_-NTs (Ti/B-TiO_2_-NTs) layer was fabricated as an interlayer for making a more stable PbO_2_ anode. Additionally, sodium dodecyl sulfate (SDS) was adopted to modify the PbO_2_ active layer via electrochemical deposition. The physicochemical and electrochemical properties of this novel PbO_2_ anode were characterized. The electrochemical oxidation behavior of MB on the different types of PbO_2_ anodes was investigated fully. The degradation efficiencies of MB were also evaluated under various experimental parameters. Radical trapping experiments were performed to further explore the role of ·OH and ·SO_4_^−1^ in the degradation of MB.

## 2. Results and Discussion

### 2.1. Morphology and Phase Analysis

**Figure 1:**
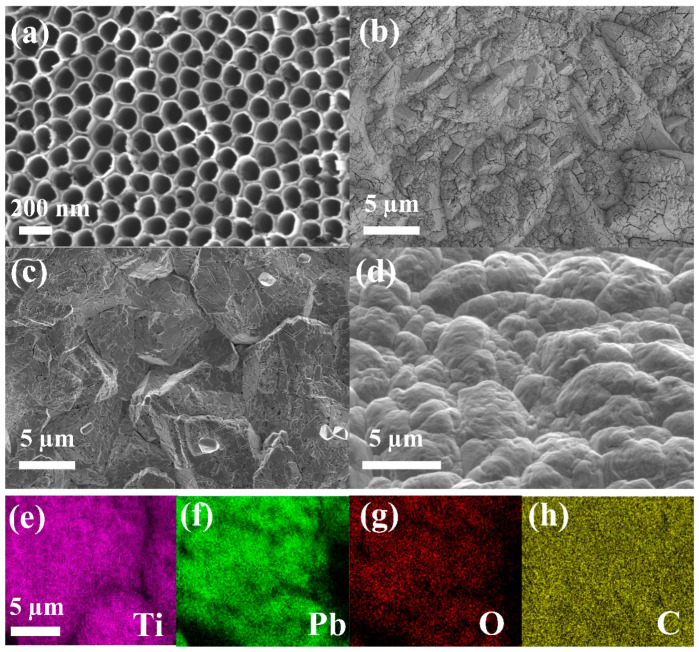
SEM images of the surface morphology of (**a**) Ti/B-TiO_2_-NTs, (**b**) Ti/TiO_2_-NTs/PbO_2_, (**c**) Ti/B-TiO_2_-NTs/PbO_2_, and (**d**) Ti/B-TiO_2_-NTs/PbO_2_-SDS electrode; EDS elemental mappings for Ti (**e**), Pb (**f**), O (**g**), and C (**h**) on the Ti/B-TiO_2_-NTs/PbO_2_-SDS electrode surface.

The morphologies of the different electrodes were observed using SEM, and the results are shown in Figure 1 and Figure S1. From Figure S1, it can be seen that the high-ordered TiO_2_-NTs without Ti^3+^ self-doping possess a large specific volume with an average diameter of approximately 140 nm. Figure 1a shows the Ti^3+^ self-doping TiO_2_-NTs after electrochemical reduction treatment, where the nanostructure of TiO_2_NTs has not been destroyed, similar to that shown in Figure S1. Figure 1b–d display SEM images of three types of electrodes: Ti/TiO_2_-NTs/PbO_2_ without Ti^3+^ self-doping, Ti^3+^ self-doping Ti/B-TiO_2_-NTs/PbO_2_, and Ti/B-TiO_2_-NTs/PbO_2_-SDS electrodes. From Figure 1b, we can see that the surface of the coated PbO_2_ layer appears coarse with noticeable cracks on the surface of Ti/TiO_2_-NTs without Ti^3+^ self-doping, which could result in the permeation of electrolytes and reduce electrode stability. Figure 1c shows the morphology of deposited PbO_2_ on Ti^3+^ self-doping TiO_2_-NTs. Compared with the Ti/TiO_2_-NTs/PbO_2_ electrode, the surface of the Ti/B-TiO_2_-NTs/PbO_2_ electrode still exhibits the pyramid structures, but it becomes smoother and the cracks have disappeared. The surface structure of PbO_2_ after SDS addition on Ti^3+^ self-doping TiO_2_-NTs is the smoothest, most uniform, and extraordinarily compact among these three electrodes (Figure 1d); the pyramid structures disappeared, and the grain size significantly decreased. This result indicates that adding SDS can help obtain a surface coating that is uniform, compact, and smooth. This may be because of SDS’s ability to disperse the Pb^2+^ evenly in the solution [15]. Furthermore, this result also suggests that the surface morphology of the Ti/TiO_2_-NTs/PbO_2_ electrode is simultaneously affected by both Ti^3+^ self-doping and SDS. The elemental mapping of the Ti/B-TiO_2_-NTs/PbO_2_-SDS electrode further confirmed the homogeneous distribution of Pb, O, Ti, and C elements in the Ti/B-TiO_2_-NTs/PbO_2_-SDS electrode (Figure 1e).

**Figure 2:**
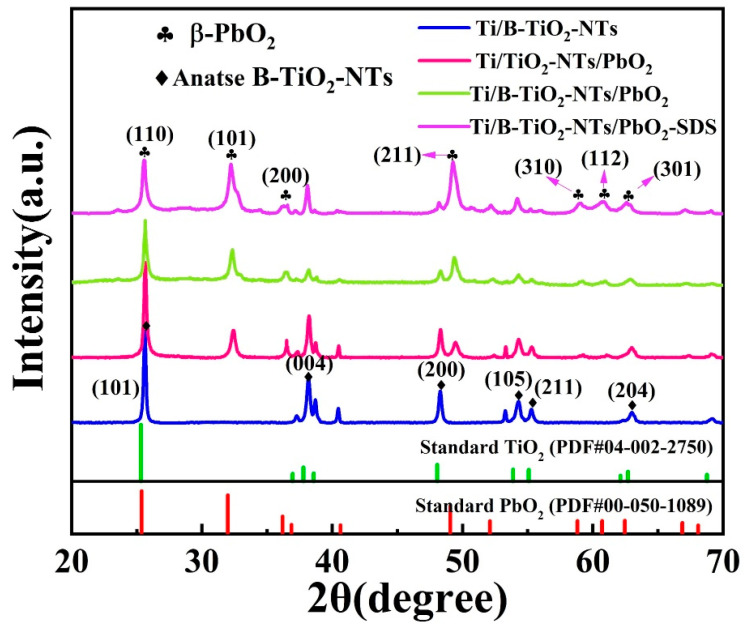
XRD patterns of Ti/B-TiO_2_-NTs/PbO_2_-SDS electrode.

The typical XRD patterns of three self-made electrodes are shown in Figure S2 and Figure 2. As shown in Figure S2, all the diffraction peaks of Ti^3+^ self-doping Ti/B-TiO_2_-NTs were identically in agreement with those of the no-doping Ti/TiO_2_-NTs, indicating no change in the anatase phase of the Ti^3+^ self-doping TiO_2_NTs within their crystal structure during electrochemical reduction process.

For the XRD pattern of the PbO_2_ electrode in Figure 2, the diffraction peaks for all samples located at 2θ = 25.56°, 32.28°, 36.23°, 49.11°, 58.96°, 60.76°, and 62.53° can be attributed to the (110), (101), (200), (211), (310), (211), and (301) planes of β-PbO_2_, respectively, which matched with the standard diffraction peaks of the JCPDS card (No. 76-0564). By comparison with three PbO_2_ electrodes, it can be clearly seen that the diffraction intensities of the (101), (211), and (301) planes of Ti/B-TiO_2_-NTs/PbO_2_ and Ti/B-TiO_2_-NTs/PbO_2_-SDS electrodes increased, while the diffraction intensities of the (110), (200), and (211) planes decreased. This means that Ti^3+^ self-doping and the addition of SDS do not alter the crystal structure but would affect the crystal plane orientation of *β*-PbO_2_ [16]. It can be argued that Ti^3+^ self-doping or SDS addition will change the electric double-layer structure, altering the growth mechanism of the PbO_2_ phase and electrodeposition kinetic process on the anode surface, which could inhibit the growth of (110) and (200). The average grain sizes of three different PbO_2_ electrodes were calculated using the Debye–Scherrer formula, as shown in Table S1. The results indicate that the average particle sizes of the *β*-PbO_2_ electrode significantly decreased upon doping with Ti^3+^ and adding the anionic SDS surfactant. The small crystal particle size is favorable for forming a large specific area, which is conducive to improving the electrochemical activity of the electrode. Thus, the results from XRD are consistent with the above-mentioned result of SEM.

**Figure 3:**
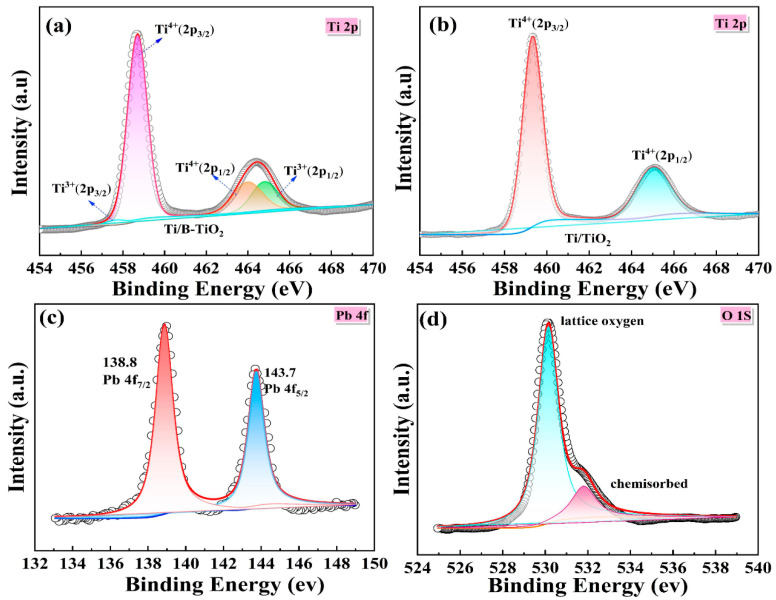
The high-resolution XPS spectra of (**a**) Pb 2p, (**b**) O 1s, and (**c**) Ti 3d of Ti/B-TiO_2_-NTs and (**d**) Ti 3d of Ti/TiO_2_-NTs.

For the purpose of analyzing the chemical composition and surface elements on the prepared PbO_2_ electrode materials, the XPS measurements were used to investigate the chemical states and elementary composition of PbO_2_ electrodes. The results are shown in Figure 3a–d. The binding energy was corrected using the C 1s peak at 284.6 eV. From the survey spectrum shown in Figure S3, characteristic binding energy peaks of Pb, Ti, C, and O elements were found on the surface of the Ti/B-TiO_2_-NTs/PbO_2_-SDS electrode. The detailed spectrum of Ti 2p is shown in Figure 3a,b. The high-resolution XPS spectra of the Ti 2p peak of Ti/B-TiO_2_ can be deconvoluted into four peaks (Figure 3a). Two binding energies of 459.14 and 465.13 eV can be assigned to Ti^4+^, while the binding energies of 457.68 and 464.79 eV are attributed to Ti^3+^ [17]. Compared to Ti/TiO_2_-NTs (Figure 3b), the peaks of Ti/B-TiO_2_-NTs show a slight red-shift to higher binding energy (Figure S4). The results indicate that Ti^3+^ and oxygen vacancies exist in the Ti/B-TiO_2_-NTs, demonstrating that the Ti^3+^ reduction is irreversible. Therefore, the conductivity of Ti/B-TiO_2_-NTs can be markedly improved. In the detailed spectrum of Pb 4f in Figure 3c, the two peaks of Pb4f corresponding to Pb^4+^ 4f_5/2_ and Pb^4+^ 4f_7/2_ are shown at around 137.08 eV and 141.97 eV, which were identified as the spectral values of PbO_2_ [18]. The deconvolution of the O 1s spectrum could also be fitted into two characteristic peaks at 528.5 and 529.3 eV (Figure 3d), which belong to lattice oxygen (O_L_) and chemisorbed oxygen (O_ads_, OH) [19].

### 2.2. Electrochemical Characterization

Figure S5a shows the CV data of the TiO_2_NTs electrodes before and after electrochemical reduction.

**Figure 4:**
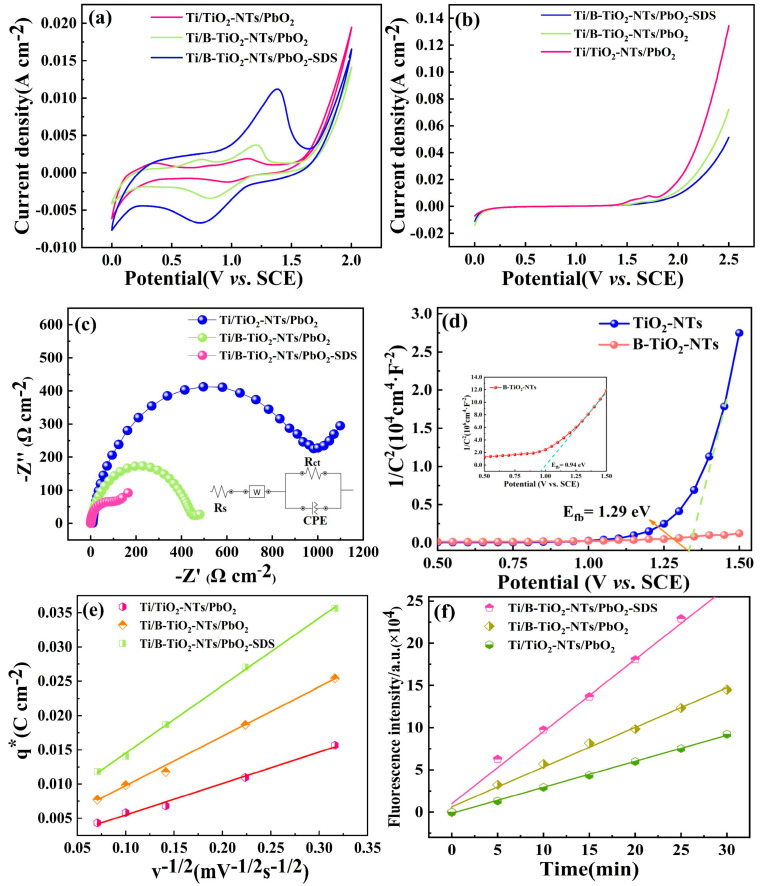
(**a**) CV curves of the prepared different PbO_2_ electrodes measured in the 0.2 M Na_2_SO_4_ solution at a scan rate of 50 mV s^−1^. (**b**) LSV of as-fabricated electrodes in the 0.2 M Na_2_SO_4_ solution at a scan rate of 50 mV s^−1^. (**c**) EIS of different PbO_2_ electrodes in the 1 M H_2_SO_4_ solution and the equivalent circuit. (**d**) Mott–Schottky analysis. (**e**) The relationship of (q*) and the reciprocal of scan rate obtained on different PbO_2_ electrodes. (**f**) Evolution of OH radical concentration of various electrodes at different periods during electrocatalytic oxidation.

The pristine TiO_2_NTs exhibited a low response current within the potential window of 0~2 V, indicating the poor conductivity of TiO_2_NTs caused by its semiconductive nature. In contrast, the Ti^3+^ self-doping Ti/B-TiO_2_-NTs presented a much higher response current in the same potential window, implying that the electrical conductivity was markedly improved. EIS was adopted to probe the conductivity of the Ti/B-TiO_2_-NTs. Figure S5b shows the Nyquist plot of Ti/TiO_2_NTs and Ti/B-TiO_2_-NTs electrodes. It can be clearly observed that the Ti/B-TiO_2_-NTs show a smaller semicircle radius on Nyquist plots than the pristine Ti/TiO_2_NTs, indicating that the resistance of the Ti/B-TiO_2_-NTs electrode was significantly decreased. This is further proof that the introduction of Ti^3+^ ions into TiO_2_NTs can improve electrical conductivity [17]. It demonstrated that Ti^3+^ self-doping can significantly improve the electrochemical performance of the TiO_2_NTs. The CV curves were obtained for the different PbO_2_ electrodes under a 0.2 M Na_2_SO_4_ solution at a scanning rate of 50 mV s^−1^. As observed from CV curves of the various PbO_2_ electrodes (Figure 4a), all PbO_2_ electrodes exhibited similar shapes with a pair of redox peaks, which corresponded to the reduction–oxidation reactions between Pb (II) and Pb (IV) at the electrode interface [20]. However, the peak oxidation current density of the Ti/B-TiO_2_-NTs/PbO_2_-SDS electrode was higher than that of the other two PbO_2_ electrodes, which implies that the introduction of Ti^3+^ in the interlayer of Ti/TiO_2_-NTs and the addition of SDS in the active layer of PbO_2_ played a role in improving the electrode activity. It also means that the Ti/B-TiO_2_-NTs/PbO_2_-SDS electrode has a greater number of active sites. Therefore, the Ti/B-TiO_2_-NTs/PbO_2_-SDS electrode is much more suitable for the electrocatalytic degradation of MB than other electrodes.

The LSV of the different PbO_2_ electrodes was examined in 0.2 M Na_2_SO_4_ at a scan rate of 50 mV s^−1^ to investigate the anodic oxygen evolution potential (OEP). As presented in Figure 4b, the Ti/B-TiO_2_-NTs/PbO_2_-SDS electrode achieved the highest OEP of 2.11 V vs. SCE. This result might be due to the small crystal structure and the synergistic effect of SDS and Ti^3+^. As is known, the oxygen evolution reaction is the main competitive side reaction on the anode surface (Equation (1)), which can lead to increased energy consumption and decreased oxidation efficiency.

∙OH_ad_ +H_2_O→O_2_ +3H^+^ +3 e^−1^ (1)

Therefore, the highest OEP would favor the Ti/B-TiO_2_-NTs/PbO_2_-SDS electrode and exhibit a stronger oxidation ability for the electrochemical oxidation of organics [21]. A higher OEP value means a substantial amount of ·OH could be generated and accelerate the electrochemical degradation of contaminants, leading to a stronger electrocatalytic ability with higher current efficiency.

To further evaluate the electrochemical performance of all PbO_2_ electrodes, EIS was carried out to investigate the electrical conductivity and charge-transfer resistance through the electrolyte of different electrodes. The Nyquist plots for these electrodes obtained in 0.5 M H_2_SO_4_ solution with a frequency range of 10,000 to 0.01 Hz are displayed in Figure 3c. As shown in Figure 4c, the semicircle arc of the Ti/B-TiO_2_-NTs/PbO_2_-SDS electrode in the high-to-medium frequency range of Nyquist plots was smaller than that of the other PbO_2_ electrodes, suggesting that the charge-transfer capacity of modified Ti/B-TiO_2_-NTs/PbO_2_ electrode is dramatically enhanced. The suitable equivalent circuit model shown in the inner part of Figure 4c and the EIS simulation results were obtained by fitting experimental data as shown in Table S2, in which R_s_ is used to present the solution resistance and R_ct_ and W correspond to charge-transfer resistance and Warburg resistance, respectively. The constant phase elements (CPE) refer to the real double-layer capacitors. It can be seen that the R_ct_ of the Ti/B-TiO_2_-NTs/PbO_2_-SDS electrode was smaller than that of the other PbO_2_ electrodes, indicating its better electrical conductivity and high charge-transfer rate [22]. The significantly promoted charge-transfer performance of the Ti/B-TiO_2_-NTs/PbO_2_-SDS electrode may be attributed to its compact β-PbO_2_ crystal structure and the good conductivity of the Ti^3+^ self-doping Ti/B-TiO_2_-NTs interlayer. So, the electrochemical degradation reaction can proceed more easily on this electrode. In addition, the improvement in electron-transfer efficiency of the undoped Ti/TiO_2_-NTs, the Ti^3+^ self-doping Ti/B-TiO_2_-NTs and Ti/B-TiO_2_-NTs/PbO_2_-SDS was further confirmed by the Mott–Schottky measurements at a frequency of 500 Hz in the 0.5 M H_2_SO_4_ solution. Figure 4d presents the Mott–Schottky plots of the pristine TiO_2_NTs and Ti^3+^ self-doping Ti/B-TiO_2_-NTs. It was intriguing to note that the Ti^3+^ self-doping TiO_2_NTs material displayed a substantially smaller slope of Mott–Schottky plots than the other pristine TiO_2_NTs, suggesting an increase in donor density due to the presence of Ti^3+^ (oxygen vacancies) under the electrochemical reduction, which effectively promotes the electrical conductivity and charge transport. Therefore, compared with the traditional Ti/TiO_2_-NTs-PbO_2_ electrode, the Ti^3+^ self-doping Ti/B-TiO_2_-NTs-PbO_2_ electrode with its improved electron-transfer efficiency possessed an enhanced electrochemical activity in the degradation process.

The electrochemical activity of electrode materials is strongly associated with the quantity of active sites on the surface of electrodes [23]. The voltammetric charge quantity (q*) is considered to be related to the electrochemical activity and the number of active sites of PbO_2_ electrodes. The higher q* means more accessible active sites on the anode surface. The q* value can be calculated with the following equation [24]:

q* = q_o_* + kν^−1/2^ (2)

The outer charge quantity (q_o_*) is the total quantity of theoretically active sites on the electrode surface, where ν is the scan rate of voltage and k is a constant. The linear relationship between q* and v^−^^1/2^ by fitting with linear correlations is shown in Figure 4e. It was noticed that the Ti/B-TiO_2_-NTs/PbO_2_-SDS electrode obtained the largest q* among the three PbO_2_ electrodes. This result indicates that the Ti/B-TiO_2_-NTs/PbO_2_-SDS electrode would exhibit good electrocatalytic performance for the electrochemical degradation of contaminants, which can be attributed to its larger specific surface area and a large number of active sites.

The hydroxyl radical (·OH) generation capacity of PbO_2_ can also be used to evaluate its electrochemical catalytic performance [25]. Hence, the ·OH radical generation ability of different electrodes was tested using fluorescent spectrometry. Terephthalic acid was used as a probe for ·OH radicals, generating a highly fluorescent product of 2-hydroxyterephthalic acid. So, the amounts of the generated ·OH radicals can be reflected by the fluorescence intensity of 2-hydroxyterephthalic acid. A linear relationship between fluorescence intensity and electrolysis time is shown in Figure 4f. According to Figure 4f, the fluorescence intensity on the Ti/B-TiO_2_-NTs/PbO_2_-SDS electrode is higher than that of the other two PbO_2_ electrodes, which implies that it has the strongest ·OH radical generation ability. This demonstrates once again that the Ti/B-TiO_2_-NTs/PbO_2_-SDS electrode exhibits higher electrocatalytic activity than the pristine PbO_2_ electrode for degrading organic pollutants.

### 2.3. Electrochemical Oxidation Degradation of Methylene Blue (MB)

#### 2.3.1. Effects of Different Anodes

**Figure 5:**
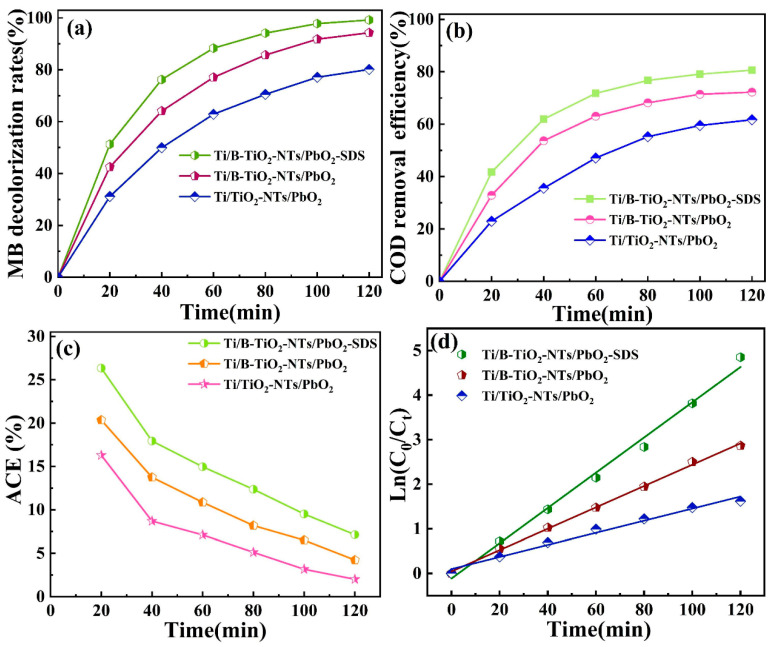
The comparisons of different electrodes: (**a**) MB decolorization efficiency, (**b**) COD removal efficiency, (**c**) ACE and (**d**) kinetic analysis curves. Conditions: current density: 40 mA cm^−2^, initial MB concentration: 30 mg L^−1^, initial pH = 3, T = 25 °C, Na_2_SO_4_ concentration = 0.2 M.

As the core component of the electrochemical oxidation process, anode materials play a key role that strongly depends on the efficiency of organic wastewater degradation. The electrocatalytic oxidation abilities of the three different PbO_2_ electrodes were investigated for the degradation of 30 mg L^−1^ MB with an applied current density of 40 mA cm^−2^ at 25 °C. In Figure 5a, it can be found that an MB decolorization rate of up to 99.7% was achieved by the Ti/B-TiO_2_-NTs/PbO_2_-SDS electrode during 120 min electrolysis, which was higher than that of the Ti/TiO_2_-NTs/PbO_2_ and Ti/B-TiO_2_-NTs/PbO_2_ electrodes. From Figure 5b, it is easy to observe that the Ti/B-TiO_2_-NTs/PbO_2_-SDS electrode exhibited the highest COD removal efficiency among the three prepared electrodes during 120 min, indicating its higher electrocatalytic oxidation capacity compared to the Ti/TiO_2_-NTs/PbO_2_ and Ti/B-TiO_2_-NTs/PbO_2_ electrodes.

In addition, the pseudo-first-order kinetics of the degradation of MB concentration with time was analyzed, and the results are shown in Figure 5c. From Figure 5c, the semilogarithmic plot was in good agreement with the pseudo-first-order kinetics, and the highest rate constant (**k**) was obtained under the optimal conditions by employing the enhancement of Ti/B-TiO_2_-NTs/PbO_2_-SDS electrode. The pseudo-first-order kinetic constant of the Ti/B-TiO_2_-NTs/PbO_2_-SDS electrode was 0.03956 min^−1^, which was approximately 3.18 times faster than that of the Ti/TiO_2_-NTs/PbO_2_ electrode (0.01254 min^−1^). The results were attributed to the large specific surface area and more active sites of the Ti/B-TiO_2_-NTs/PbO_2_-SDS electrode. Furthermore, the average current efficiency (ACE) of the different PbO_2_ electrodes was investigated under different degradation time period, and the results are exhibited in Figure 5d. The Ti/B-TiO_2_-NTs/PbO_2_-SDS electrode exhibits a higher level of ACE than that of the Ti/TiO_2_-NTs/PbO_2_ and Ti/B-TiO_2_-NTs/PbO_2_ electrodes during the whole degradation of MB, which indicated its high-efficiency performance and better energy conservation. Table S3 lists the COD removal efficiency of other types of PbO_2_ electrodes reported in the literature. By comparison, obviously, the Ti/B-TiO_2_-NTs/PbO_2_-SDS electrode had better activity.

The above results demonstrate that the Ti/B-TiO_2_-NTs/PbO_2_-SDS electrode possesses the best electrochemical activity for the degradation of MB compared to the other two electrodes. This can be attributed to its stronger hydroxyl radical generation rate, more electrochemical active sites, and smaller charge-transfer resistance than the other two electrodes.

#### 2.3.2. Effect of Current Density

**Figure 6:**
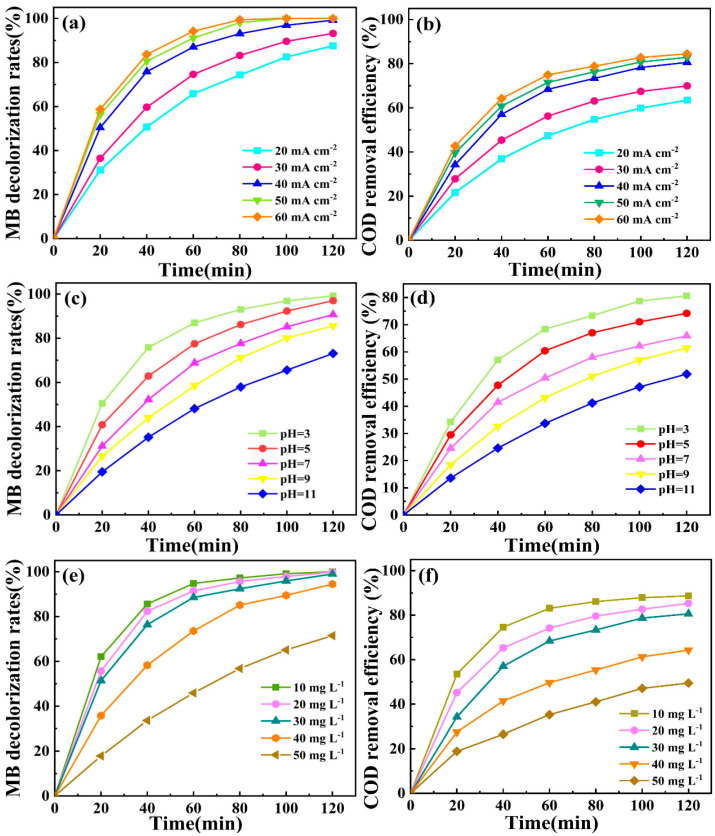
Effects of different conditions on the MB decolorization and COD removals: (**a**,**b**) the effect of applied current density; (**c**,**d**) initial pH and (**e**,**f**) initial concentration.

The current density is an important influencing parameter in the electrochemical oxidation for the degradation of MB [26,27]. Therefore, it is necessary to investigate the influence of current density on the degradation of MB. The effect of current density on the Ti/B-TiO_2_-NTs/PbO_2_-SDS electrode for the degradation of MB was investigated at pH = 3, the MB concentration of 30 mg L^−1^, and the electrolyte Na_2_SO_4_ concentration of 0.2 M. It can be found from Figure 6a that the removal efficiency of MB increased from 87.60% to 99.9% when the current density changed from 20 to 60 mA cm^−2^. In addition, the degradation of MB at different current densities was fitted by a pseudo-first-order kinetic model, and the kinetics parameters are shown in the inset of Figure S6a. The corresponding *k* value also increased with the increase in current density. As displayed in Figure 6b, the COD degradation efficiencies also gradually increased with the increase in current densities. The reason for this phenomenon is that a high yield of ·OH radicals can be formed at a high current density [28,29]. However, according to Figure 5a,b, there was a slight increase in decolorization efficiency and COD removal efficiency when the current density was more than 50 mA cm^−2^. This could be attributed to the more serious oxygen evolution side reaction, leading to a decreased current efficiency [30]. Therefore, 40 mA cm^−2^ was selected as the optimal current density for the following studies.

#### 2.3.3. Effect of Initial pH

In this work, the influence of initial pH values ranging from 3 to 11 on the degradation of MB is shown in Figure 6c,d. The results revealed that the decolorization ratio of MB and COD in an acidic medium is higher than that in neutral and alkaline environments. The highest removal ratio of MB and COD is achieved at pH= 3. The kinetic rate constant (k) value at pH = 3 is 4.02 times higher than that at pH = 11 (Figure S6b). In an acid medium, H^+^ can inhibit the decomposition reaction of ·OH into oxygen [31,32]. In order to keep a high content of hydroxyl radicals, pH = 3 was selected for further research.

#### 2.3.4. Effect of Initial Concentration

It is meaningful to investigate the effect of the initial concentration of MB in the electrochemical oxidation process because MB exists in industrial wastewater with a wide range of concentrations.

The influence of the initial MB concentration on the degradation rate is shown in Figure 6e,f. As observed in Figure 5e,f, the MB decolorization ratio significantly decreased from 99.98% to 71.45% and the removal efficiency of COD decreased from 88.70% to 49.46% with an increase in initial MB concentration. The kinetic constant (k) increased as the concentration decreased (Figure S6c). The possible reason for this phenomenon was that more intermediates were generated and accumulated in large quantities on the electrode surface with the increase in the initial concentration during the degradation of MB. This hindered the formation of ·OH and indirect oxidation, leading to a decrease in the degradation of MB [33]. The kinetic constant (k) gradually increased from 0.01145 min^−1^ to 0.05495 min^−1^ as the concentration decreased (Figure S6c).

#### 2.3.5. Effect of Electrolyte Concentration

**Figure 7:**
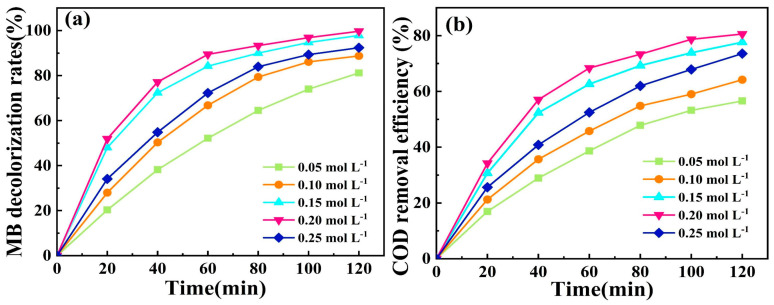
The effect of supporting electrolyte (Na_2_SO_4_) concentration on (**a**) MB removal efficiency with time and (**b**) COD removal efficiency with time on Ti/B-TiO_2_-NTs/PbO_2_-SDS electrode (initial pH: 3; initial MB concentration: 30 mg L^−1^; current density: 40 mA cm^−2^).

The supporting electrolyte concentration promotes the conductivity of the degradation solution and is an essential factor in the electrochemical degradation process for reducing energy consumption. The effect of Na_2_SO_4_ concentration from 0.05 to 0.25 M on the MB degradation efficiency was investigated at different supporting electrolyte concentrations. As displayed in Figure 7a,b, the MB decolorization and COD removal efficiency initially increase and then decrease. A similar tendency can be observed for the kinetic constant (k) (Figure S6d).

This phenomenon is ascribed to the fact that Na_2_SO_4_ can improve the conductivity of the solution with an increase in electrolyte concentration. However, when the concentration of Na_2_SO_4_ is too high, the electrode would be coated with adsorbed SO_4_^2−^ and occupy the active site on the electrode surface, thus leading to a reduction in the generation of oxidizing radicals during electrochemical oxidation [34,35].

### 2.4. Degradation Mechanism of MB Wastewater

#### 2.4.1. The UV-Vis Absorption Spectra Analysis

**Figure 8:**
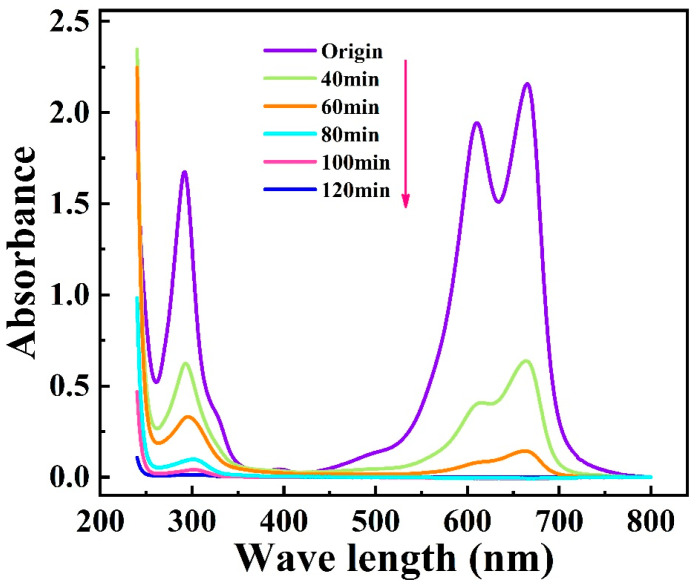
The variation in MB absorption spectra with the reaction time.

The UV–visible absorption spectra were measured at different times, and the results are shown in Figure 8. As shown in Figure 8, the original UV-vis absorption peaks of MB had three distinct absorption peaks at 292, 602 and 665 nm, respectively [36,37]. With the increase in degradation time, the characteristic peaks at 610 nm and 664 nm decreased. This may be ascribed to the N-demethylation of MB [37]. More importantly, no obvious characteristic peaks of MB were found after a degradation period of 120 min, illustrating that MB had been completely degraded. Therefore, the as-prepared Ti/B-TiO_2_-NTs/PbO_2_-SDS electrode had an efficient capacity in the degradation of MB.

#### 2.4.2. Trapping Experiments of Radicals

**Figure 9:**
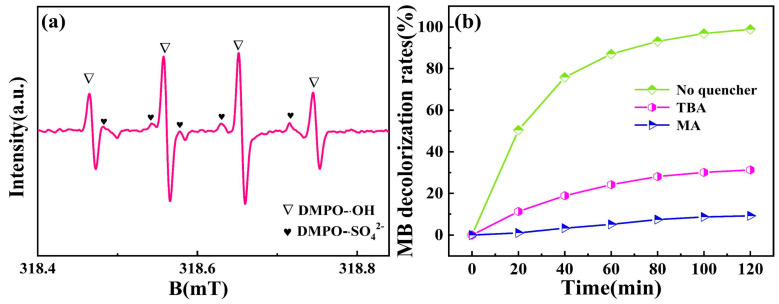
(**a**) ESR spectra of the free radicals in electrochemical oxidation process with Ti/B-TiO_2_-NTs/PbO_2_-SDS electrode. (**b**) Degradation effect of MB under different quencher conditions.

Electron spin resonance (ESR, Hitachi JES-FA200) was employed to trap the possible free radicals of ·OH and sulfate (·SO_4_^−1^) generated in the electrochemical system using a Ti/B-TiO_2_-NTs/PbO_2_-SDS electrode. 5, 5-dimethyl-1-pyrroline-N-oxide (DMPO) as spin-trapping agent was used to capture possible free radicals generated in the electrochemical system. As shown in Figure 9a, significant signals of DMPO-·OH and DMPO-·SO_4_^−1^ can be observed, suggesting that the reactive groups produced were ·OH and ·SO_4_^−1^ in the electrochemical oxidation process.

To further explore the role of ·OH and ·SO_4_^−1^ in the degradation of MB, radical trapping experiments were performed to track the contribution of ·OH and ·SO_4_^−1^ radicals during the electrocatalytic reaction. Methanol (MA) and Tertbutanol (TBA) were used as free radical scavengers. MA could capture both ·OH and ·SO_4_^−1^ radicals, while TBA was only adopted as the scavenger of ⋅OH radicals [38]. Figure 9b clearly indicates that ·OH played a primary role in MB degradation. From Figure 9b, it can be observed that the efficiency of MB degradation decreased significantly when MA and TBA were added to the wastewater containing MB. The high suppression ratios indicate that the indirect oxidation process is mainly responsible for the degradation of MB.

### 2.5. Electrode Stability and Recyclability

**Figure 10:**
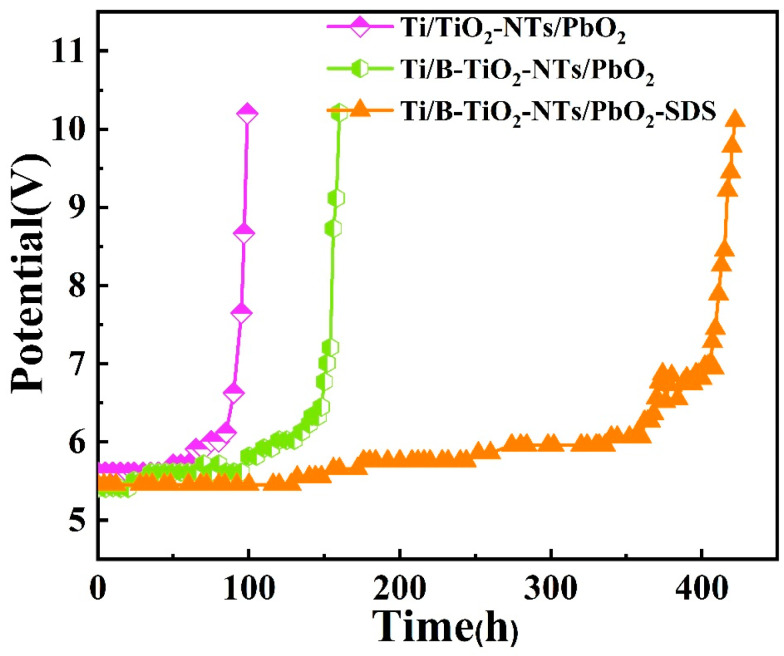
The accelerated life test of different electrodes in 2 M H_2_SO_4_ solution at 1.0 A cm^−2^ current density and temperature at 60 °C.

The stability of the electrodes is a significant factor in evaluating the electrochemical oxidation performance of the electrodes. It can be inferred from the data displayed in Figure 10 that the Ti/B-TiO_2_-NTs/PbO_2_-SDS electrode possessed a longer service life (442 h), which was 4.49 times longer than that of the Ti/TiO_2_-NTs/PbO _2_ electrode (99 h). This result indicates that the stability of Ti/TiO_2_-NTs/PbO_2_ was significantly improved after introducing Ti^3+^ and SDS. Meantime, the electrochemical recycling test was also carried out under the optimal conditions. Figure S7 shows that the MB removal efficiency slightly decreased from 99.2 to 96.1% after 20 consecutive cycles. Such a small reduction in MB removal percentage demonstrates the high chemical stability and recyclability for electrochemical oxidation, which can be attributed to the compact structure of PbO_2_ film with the introduction of Ti^3+^ and SDS. These results indicate that the Ti/B-TiO_2_-NTs/PbO_2_-SDS is a promising electrode anode material for the treatment of textile wastewater.

## 3. Experimental

### 3.1. Materials and Reagents

All chemical reagents were analytical-grade and provided by Sinopharm Chemical Regent Co. Ltd., Shanghai, China. The solutions were prepared using ultrapure water.

### 3.2. Preparation of Ti^3+^ Self-Doping Ti/B-TiO_2_-NTs/PbO_2_-SDS Electrode

**Scheme 1:**
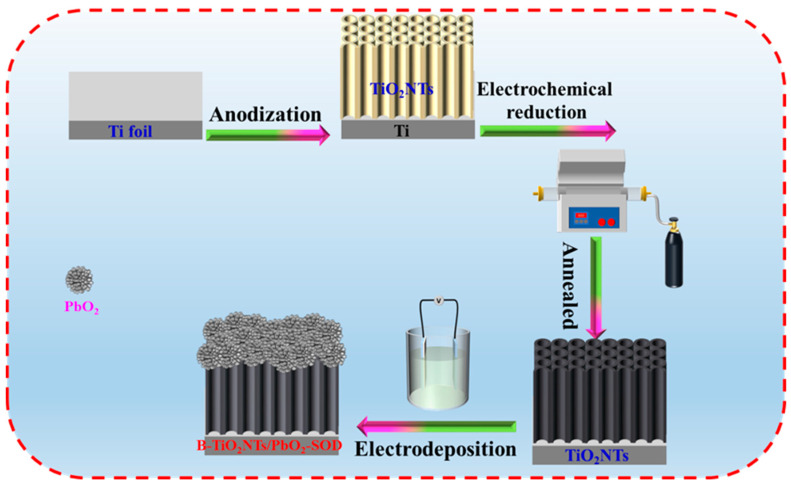
The preparation procedure of the Ti/B-TiO_2_-NTs/PbO_2_-SDS electrode.

As illustrated in Scheme 1, the Ti^3+^ self-doping Ti/B-TiO_2_-NTs/PbO_2_-SDS electrode was prepared by using two main steps.

(1) Fabrication of the Ti^3+^ self-doping Ti/B-TiO_2_-NTs middle layer on the Ti substrate.

The method for preparing the Ti^3+^ self-doping Ti/B-TiO_2_-NTs material was consistent with our previous work [39]. Firstly, a Ti sheet (1.0 cm × 2.0 cm × 0.8 cm) was mechanically polished using different sizes of the abrasive papers in sequence and then was cleaned ultrasonically. Afterward, the cleaned Ti sheet was chemically etched in a mixture of HF and ethylene glycol for several minutes. The pre-treated Ti sheet was electrochemically oxidized at 60 V for 7 h to obtain as-grown nanotube arrays. For the fabrication of Ti^3+^ self-doping Ti/B-TiO_2_-NTs, Ti/TiO_2_-NTs were electrochemically reduced in 1 M (NH_4_)_2_SO_4_ solution under a constant current (5 mA cm^−2^) for 10 min with Ti/TiO_2_-NTs and platinum foil serving as cathode and anode, respectively. Then, it was calcined at 550 °C in a muffle furnace for 2 h under Ar condition. Ti^3+^ self-doping Ti/B-TiO_2_-NTs were prepared to obtain the intermediate layer. The reduced electrode was labeled as Ti/B-TiO_2_-NTs.

(2) Deposition of the PbO_2_ coating on the middle layer

Afterward, the Ti^3+^ self-doping Ti/B-TiO_2_-NTs/PbO_2_-SDS electrode was prepared by the electrodeposition method in an electrolyte containing 0.1 M HNO_3_, 0.5 M Pb(NO_3_)_2_ 10 mg L^−1^ SDS and 0.02 M NaF under 20 mA cm^−2^ for 1 h at 60 °C. For comparison, the Ti/B-TiO_2_-NTs/PbO_2_ and Ti/TiO_2_-NTs/PbO_2_ without Ti^3+^ self-doping electrodes were prepared using the same electrodeposition method.

### 3.3. Characterization

The surface morphologies and crystallographic structures of the as-fabricated electrodes were observed by field emission scanning electron microscopy (SEM, ZEISS, Sigma 300, Sigma Aldrich, St. Louis, MO, USA) and X-ray diffraction (XRD, PHILIPS, Amsterdam, The Netherlands, X’Pert PRO). The chemical composition and valence state of the electrode were determined by X-ray photoelectron spectroscopy (XPS, Thermo, Waltham, MA, USA, ESCALAB 250). In addition, a UV-vis spectrophotometer (UV-vis, Shimadzu, Kyoto, Japan, UV-3600Plus) analyzer was used to determine the various MB concentrations during different degradation processes.

### 3.4. Electrochemical Measurement

The studies of electrochemical performances were performed on an electrochemical workstation (Metrohm, Autolab302N, Utrecht, The Netherlands) with a three-electrode cell. The as-fabricated PbO_2_ electrode, platinum foil and saturated calomel electrode (SCE) were used as the working electrode, auxiliary electrode and reference electrode, respectively. Cyclic voltammetry (CV) in a 0.2 M Na_2_SO_4_ solution was utilized for analyzing the oxidative properties of all different electrodes. Linear sweep voltammetry (LSV) was performed from 0 to 2.5 V with a scan speed of 10 mV s^−1^. Electrochemical impedance spectroscopy (EIS) was implemented in a frequency range of 100 kHz to 10 MHz with a potential amplitude of 5 mV. The accelerated service lifetime tests of all different electrodes were performed at a high current density of 1 A·cm^−2^ in 2 M H_2_SO_4_. The electrode was deactivated when the cell voltage reached 10 V higher than the initial voltage.

### 3.5. Electrocatalytic Degradation

MB was selected as the simulated pollutant because it is widely used in printing, the dyeing industry and coloring paper [13,40]. During the process of experiments, a model dye wastewater of MB was electrochemically oxidized in a 100 mL beaker with a rotor stirring function. In the electrolytic cell, the prepared electrode (2 cm × 3 cm) was used as an anode, and a same-sized Ti sheet was used as the cathode. The liquid samples were drawn from the reactor every 20 min and analyzed, including the removal of MB through a UV–vis spectrophotometer and the variation in chemical oxygen demand (COD) by using a COD analyzer (Lianhua Tech. Co. Ltd., Shenzhen, China). The absorbance of MB at different times was monitored by using the UV-vis spectrophotometer analyzer at 240–800 nm. The decolorization rate (η_MB_) was calculated by the following formula [41]:

(3) $$\eta_{\mathrm{MB}}=\frac{A_{0\;}-{\;A}_{t}}{A_{t}}\times 100\%$$

A_0_ and A_t_ are the absorbance of MB at the initial and different times (t).

The COD removal efficiency (η_COD_) was calculated by the following formula [42]:

(4) $$\eta_{\mathrm{COD}\;}=\frac{{\mathrm{COD}}_{0}\;-\;{\mathrm{COD}}_{t}}{{\mathrm{COD}}_{0}}\;\times \;100\%$$

COD_0_ is the COD of the initial concentration, and COD_t_ is the COD at a given time t.

The average current efficiency of different current densities was calculated using Equation (3) [43]:

(5) $$\mathrm{ACE}=\frac{{(\mathrm{COD}}_{0}-\;{\mathrm{COD}}_{t\;})\cdot F\cdot V_{l}}{8I\cdot ∆t\cdot 1000}\times 100\%$$

where COD_0_ and COD_t_ are the chemical oxygen demand at time 0(s) and t(s), respectively; the Faraday constant F is equal to 96,487 C·mol^−1^; V_L_ is the volume of electrolyte solution (L); △_t_ is the degradation time (s); and I is the current intensity (A). The electrochemical degradation of MB was found to well fit pseudo-first-order kinetics [44,45]:

(6) $$\ln(\frac{C_{0}}{C_{t}})=\mathrm{kt}$$

where C_0_ is the initial MB concentration, C_t_ is the concentration of MB at the given time t, and k is the kinetic rate constant.

The hydroxyl (·OH) radicals generated during the electrochemical processes were detected using terephthalic acid as a fluorescence probe and then analyzed using a fluorescence spectrophotometer (Cary Elipse, Agilent, Santa Clara, CA, USA) in the range of 370–520 nm (excitation wavelength: 315 nm).

## 4. Conclusions

The target Ti^3+^ self-doping black Ti/TiO_2_-NTs/PbO_2_-SDS electrode was successfully prepared through electrochemical reduction and electrodeposition methods in this study. The morphology and crystal structure were confirmed by SEM and XRD, respectively. The experimental results demonstrated that the Ti/B-TiO_2_-NTs/PbO_2_-SDS electrode possessed a more compact structure and finer grain size than the no-doping TiO_2_NTs/PbO_2_ electrode. Moreover, the introduction of the Ti^3+^ self-doping middle layer (Ti/B-TiO_2_-NTs) and doping of SDS significantly enhanced the electrochemical activity of the resulting Ti/B-TiO_2_-NTs/PbO_2_-SDS electrode. The enhanced electrocatalytic performance of the Ti/B-TiO_2_-NTs/PbO_2_-SDS electrode was due to its higher oxygen evolution potential (2.11 V), smaller charge-transfer resistance (6.74 Ω) and stronger ·OH generating ability. After 120 min of electrolysis, the MB decolorization rate and COD removal rate were 99.7% and 80.6%, respectively. Importantly, the value of ACE was significantly higher than that of the TiO_2_NTs/PbO_2_ electrode, indicating superior current efficiency and energy conservation capabilities. The electrochemical degradation of MB followed pseudo-first-order kinetics, and the kinetic constant of the Ti/B-TiO_2_-NTs/PbO_2_-SDS electrode reached 0.03958 min^−1^, which was 3.18 times higher than that of the pristine Ti/TiO_2_NTs/PbO_2_ electrode (0.01254 min^−1^). Furthermore, the accelerated lifetime tests confirmed the excellent stability of the prepared Ti/B-TiO_2_-NTs/PbO_2_-SDS electrode with a lifetime about 4.49 times longer than that of the pristine Ti/TiO_2_-NTs/PbO_2_ electrode. As a whole, the synthesized Ti/B-TiO_2_-NTs/PbO_2_-SDS electrode has the potential for application in the electrocatalytic oxidation degradation of refractory pollutants.

## Data Availability

Not applicable.

## Supplementary Materials

The following supporting information can be downloaded at: https://www.mdpi.com/article/10.3390/molecules28196993/s1, Figure S1: Ti/TiO_2_-NTs without Ti^3+^ self-doping; Figure S2: XRD patterns of Ti/TiO_2_-NTs without Ti^3+^ self-doping and Ti^3+^ self-doping Ti/B-TiO_2_-NTs; Figure S3: XPS survey spectra of the Ti/B-TiO_2_-NTs/PbO_2_-SDS electrode; Figure S4: XPS spectra of Ti 2p orbital of Ti/TiO_2_-NTs and Ti/B-TiO_2_-NTs; Figure S5: (a) Cyclic voltammograms of the Ti/B-TiO_2_-NTs and Ti/TiO_2_-NTs electrodes; (b) EIS of the Ti/B-TiO_2_-NTs and Ti/TiO_2_-NTs electrodes; Figure S6: Pseudo-first-order kinetic fitting curves at different current density (a), initial MB concentration (b), initial pH (c) and electrolyte concentration (d); Figure S7: Repetitive experiment of Ti/B-TiO_2_-NTs/PbO_2_-SDS electrode in electrochemical oxidation of MB for 120 min. Conditions: current density: 40 mA cm^−2^, initial MB concentration: 30 mg L^−1^, initial pH = 3, T = 25 °C, Na_2_SO_4_ concentration = 0.2 M; Table S1: Crystal sizes of β-PbO_2_ grains on different electrodes; Table S2: The fitted EIS parameters; Table S3: Comparison of the performance of PbO_2_ electrodes with other reported MB degradation methods; [37,46,47,48].
