# Supplementary material for: Development of SDS-Modified PbO2 Anode Material Based on Ti3+ Self-Doping Black TiO2NTs Substrate as a Conductive Interlayer for Enhanced Electrocatalytic Oxidation of Methylene Blue
Source: Molecules. 2023 Oct 9;28(19):6993. doi: 10.3390/molecules28196993 (PMC10574806; doi:10.3390/molecules28196993)
Supplement: Supplementary file 1 [file molecules-28-06993-s001.zip › molecules-2630450-supplementary.pdf]

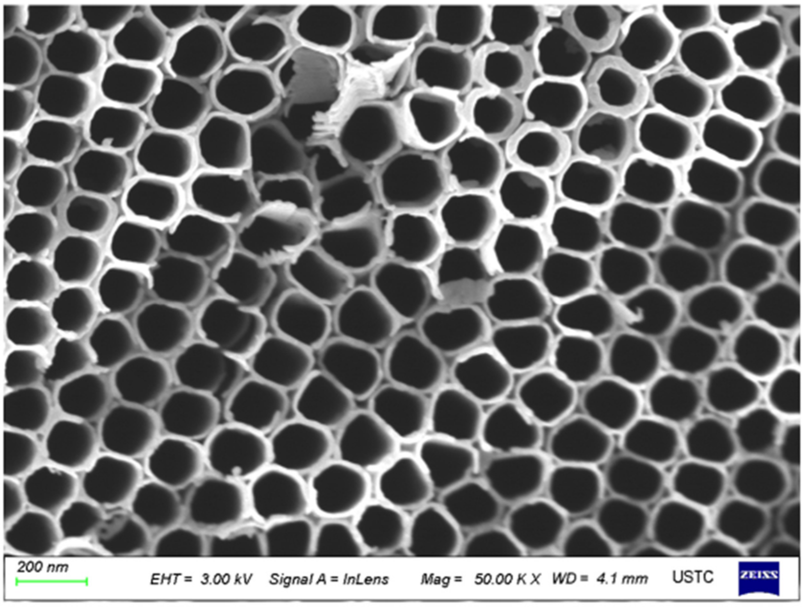

Figure. S1. No Ti^{3+} self-doping Ti/TiO_2-NTs

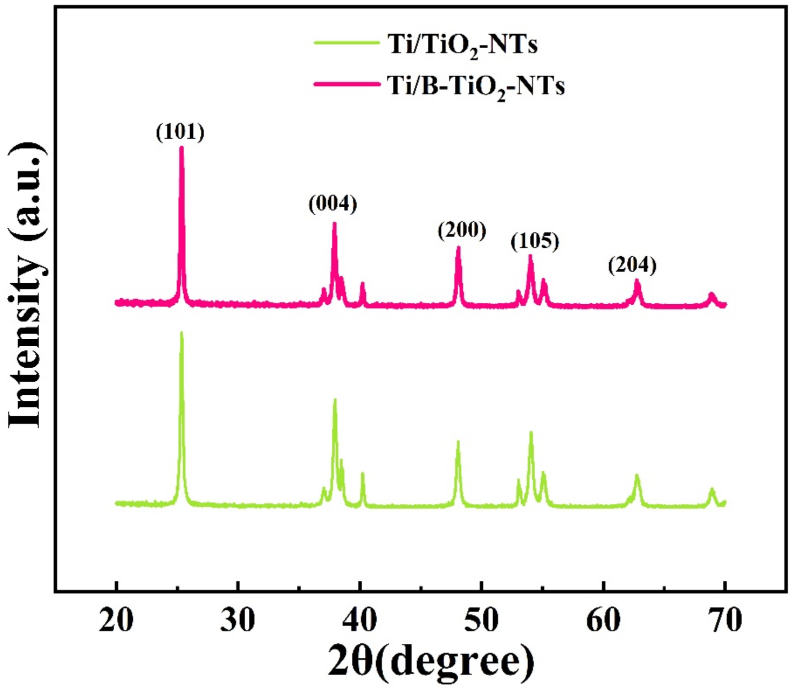

Figure. S2. XRD patterns of no Ti^{3+} self-doping Ti/TiO_2-NTs and Ti^{3+} self-doping Ti/B-TiO_2-NTs.

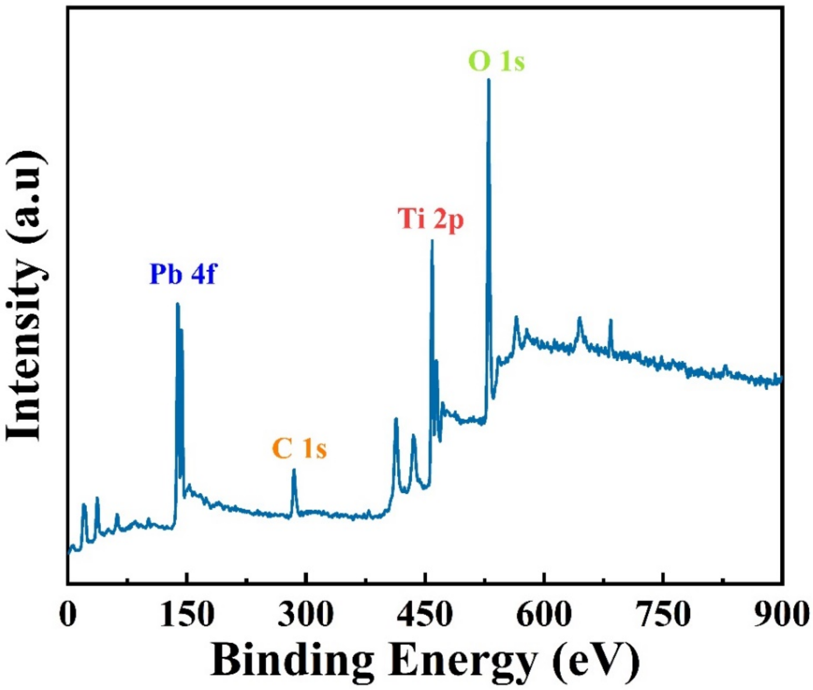

Figure. S3. XPS survey spectra of the Ti/B-TiO2-NTs/PbO2-SDS electrode.

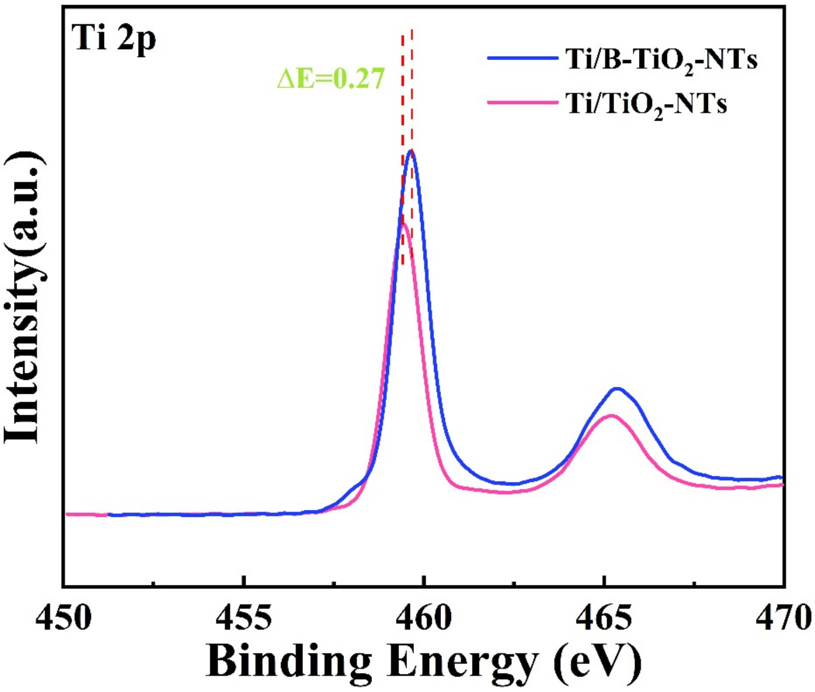

Figure. S4. XPS spectra of Ti 2p orbital of Ti/TiO2-NTs and Ti/B-TiO2-NTs.

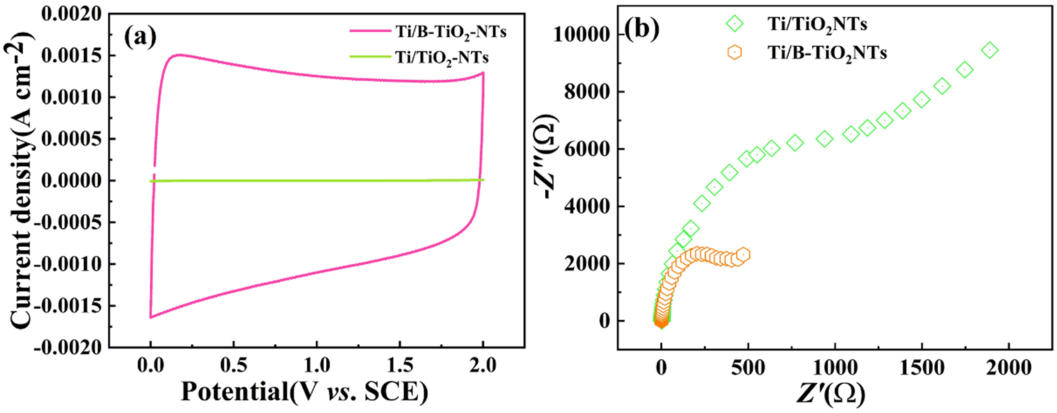

Figure. S5. (a) Cyclic voltammograms of of the Ti/B-TiO2-NTs and Ti/TiO2-NTs electrode; (b) EIS of of the Ti/B-TiO2-NTs and Ti/TiO2-NTs electrode.

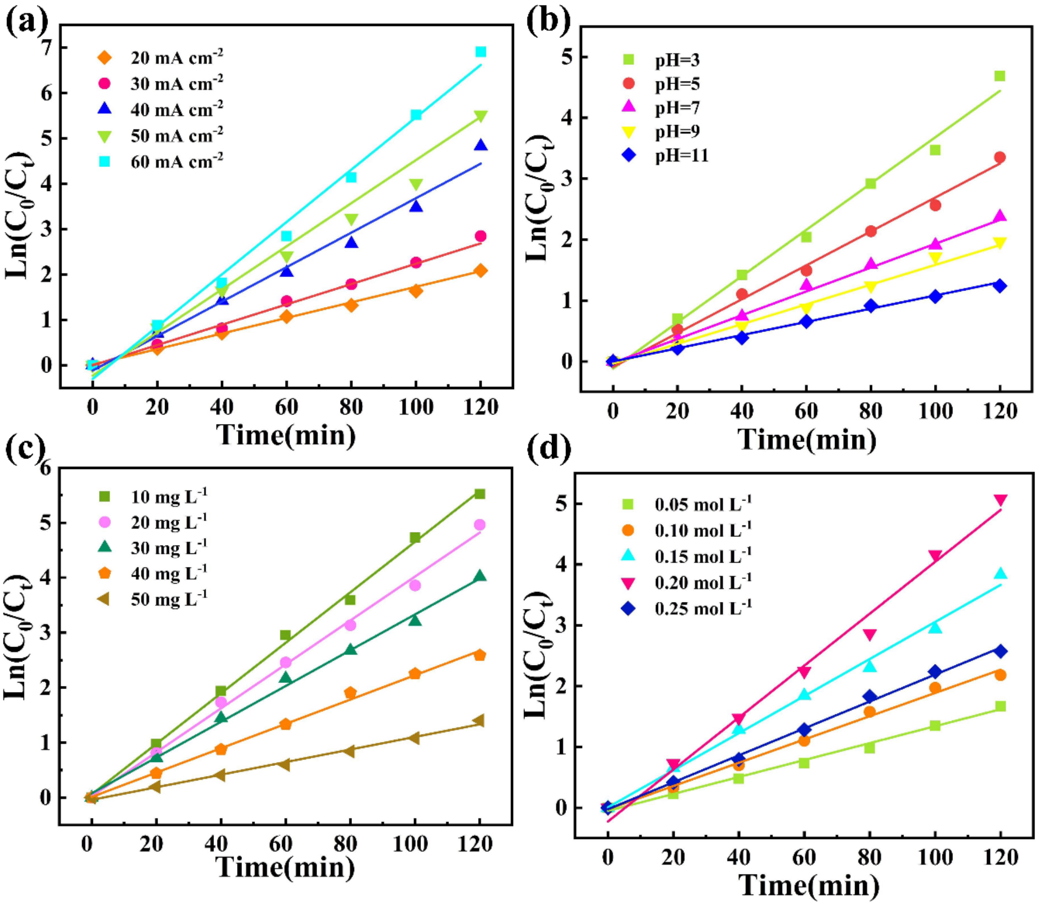

Figure. S6. Pseudo-first-order kinetic fitting curves at different current density (a), initial MB concentration (b), initial pH (c) and electrolyte concentration (d).

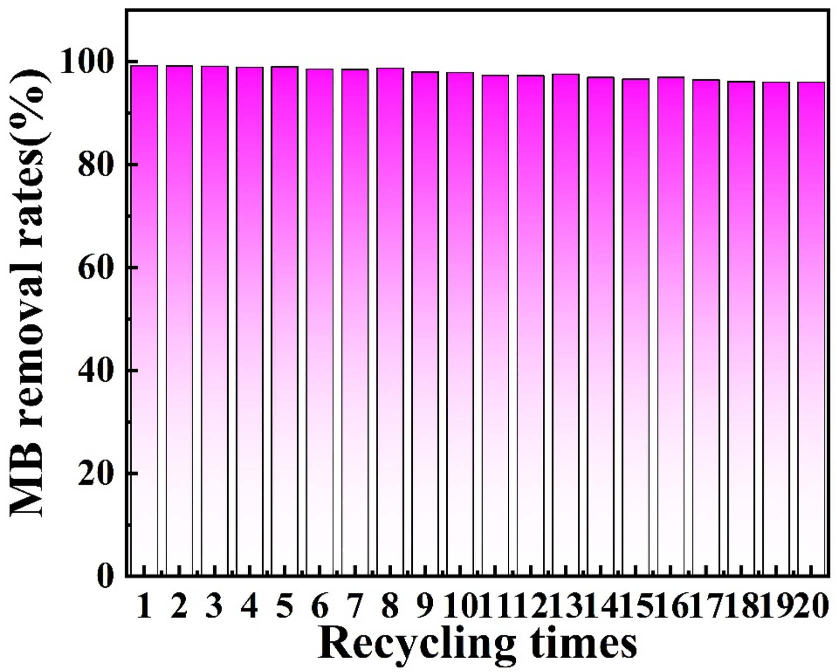

Figure. S7. Repetitive experiment of Ti/B-TiO_2-NTs/PbO_2-SDS electrode in electrochemical oxidation of MB for 120 min. Conditions: current density: 40 mA cm^{-2}, initial MB concentration: 30 mg L^{-1}, initial pH = 3, T = 25 °C, Na2SO4 concentration = 0.2 M.

Table S1. Crystal sizes of β-PbO_2 grains on different electrodes.

Electrode	Half height width (101 planes)	Intensity	Crystallite size (nm) (101 planes)
Ti/B-TiO_2-NTs/PbO_2-SDS	0.385	823	20.62
Ti/B-TiO_2-NTs/PbO_2	0.228	561	53.28
Ti/TiO_2-NTs/PbO_2	0.137	302	65.29

Table S2. The fitted EIS parameters.

Electrode	Rct (Ω cm^{-2})	Rs (mΩ cm^{-2})	CPE (mMho cm^{-2})	W (mMho cm^{-2})	n
Ti/TiO_2-NTs/PbO_2	195	363	3.42	103	0.698
Ti/B-TiO_2-NTs/PbO_2	40.7	298	5.65	126	0.845
Ti/B-TiO_2-NTs/PbO_2-SDS	6.74	145	12.9	138	0.965

Table S3. Comparison of the performance of PbO_2 electrodes with other reported degradation MB methods.

Catalytst	MB concentration	Conditions	COD orTOC removal effciency(%)	Ref
Ti/Sb_2O_3-SnO_2/Er-PbO_2	30 mg L^{-1}	0.1M Na_2SO_4,40mA/cm^2,pH=3, 120min	65.34%	[1]
PbO_2-ZrO_2	30 mg L^{-1}	0.2M Na_2SO_4,50mA/cm^2,pH=3, 120min	72.7%	[2]
S-TiO_2 NTA-PbO_2	20 mg L^{-1}	0.02M Na_2SO_4,20mA/cm^2,pH=7, 120min	49.4%	[3]
OP-10 modified PbO_2	30 mg L^{-1}	0.15M Na_2SO_4,50mA/cm^2,pH=5, 120min	70 %	[4]
Ti/B-TiO_2-NTs/PbO_2-SDS	30 mg L^{-1}	0.2M Na_2SO_4,40mA/cm^2,pH=3, 120min	80.6%	This Work

References

[1] Zhou, Y. Z. et al., Electrocatalysis enhancement of α, β-PbO_2 nanocrystals induced via rare earth Er(III) doping strategy: Principle, degradation application and electrocatalytic mechanism. *Electrochim. Acta.*2020,333,135535.

[2] Yao, Y.W., Zhao, C.M., Zhao, M.M., Wang, X., Electrocatalytic degradation of methylene blue on PbO_2-ZrO_2 nanocomposite electrodes prepared by pulse electrodeposition. *J. Hazard. Mater.* , 2013, 263,726–734.

[3] Ying, S., Li Y.D., Yao Y.W., Xia Y., Jiao M.Y., Han E.S., Electrodeposition and Catalytic Performance of Hydrophobic PbO_2 Electrode Modified by Surfactant OP-10. *ECS Journal of Solid State Science and Technology*.2021, 10,123005.

[4] Yang, C., Shang S.S., Li X.Y., Fabrication of sulfur-doped TiO_2 nanotube array as a conductive interlayer of PbO_2 anode for efficient electrochemical oxidation of organic pollutants.*J Hazard Mater.* 2021,258,118035.
